# The putative amino acid ABC transporter substrate-binding protein AapJ2 is necessary for *Brucella* virulence at the early stage of infection in a mouse model

**DOI:** 10.1186/s13567-018-0527-9

**Published:** 2018-03-29

**Authors:** Mingxing Tian, Yanqing Bao, Peng Li, Hai Hu, Chan Ding, Shaohui Wang, Tao Li, Jingjing Qi, Xiaolan Wang, Shengqing Yu

**Affiliations:** Shanghai Veterinary Research Institute, Chinese Academy of Agricultural Sciences (CAAS), Shanghai, 200241 China

## Abstract

Brucellosis is a zoonotic bacterial disease caused by *Brucella* spp. The virulence of these bacteria is dependent on their ability to invade and replicate within host cells. In a previous study, a putative gene *bab_RS27735* encoding an amino acid ABC transporter substrate-binding protein homologous to AapJ protein was found to be involved in *Brucella abortus* virulence. In this study, we successfully constructed a *bab_RS27735* deletion mutant, Δ27735. Compared with the wild-type strain, the lipopolysaccharide pattern of the mutant was not changed, but the growth ability was slightly defected in the exponential phase. In tolerance tests, sensitivity of the Δ27735 mutant to oxidative stress, bactericidal peptides or low pH was not different from that of the wild-type strain. Cell infection assay showed that the mutant was reduced survival within macrophages but could efficiently escape lysosome degradation. The results of a virulence test showed that the Δ27735 mutant was attenuated in a mouse model at the early stage of infection but recovered its virulence at the late stage of infection. Meanwhile, the development of splenomegaly and histopathological lesions was observed in mice infected with either the wild-type strain or the mutant. These results are in line with the release of IL-12p40 and TNF-α into the peripheral blood of infected mice. Besides, expression of diverse genes was up-regulated in the Δ27735 mutant, which may contribute to the reduced virulence of the mutant. These data elucidated that the *bab_RS27735* gene is necessary for *B. abortus* virulence at the early stage of infection in a mouse model.

## Introduction

Brucellosis is a zoonotic bacterial infection with *Brucella* spp. that leads to reduced animal productivity and debilitating disease in humans, which results in tremendous economic losses, especially in developing countries, and threats to public health [1, 2]. *Brucella*, as a facultative intracellular bacterium, has no classic virulence factors, such as exotoxins, cytolysins, capsules, fimbria, plasmids, lysogenic phages, drug resistant forms, antigenic variations or endotoxic lipopolysaccharide molecules. Its virulence is dependent on the ability to invade and replicate within professional or non-professional phagocytes [3, 4]. Therefore, identification of key genes involved in intracellular survival is important to elucidate the pathogenesis of *Brucella* spp.

**Figure 1:**
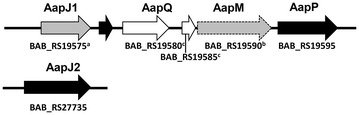
**The genetic organization of the** ***app*** **operon of** ***B. abortus***. AapJ1 and AapJ2 encode an ABC transporter substrate-binding protein; AapQ and AapM encode an ABC transporter permease; AapP encodes an ABC transporter ATP-binding protein; “a” refers to the truncated protein caused by a frameshift; “b” refers to a pseudo gene caused by a frameshift; “c” refers to the split of *aapQ* into two genes in *B. abortus* caused by a frameshift.

To date, based on a platform of the *Brucella* Bioinformatics Portal, 245 genes involved in *Brucella* virulence have been collected in a database [5]. With the development of molecular genetic techniques, more and more genes associated with *Brucella* virulence continue to be discovered [6–8], thereby offering further insight into *Brucella* pathogenesis. Transposon mutagenesis is a frequently used technique to identify virulence genes in bacterial pathogens [8–10]. In our previous study, PCR-based signature-tagged mutagenesis (STM) identified 38 novel genes involved in *Brucella* virulence, including Pyk (pyruvate kinase), which was found to be necessary for *Brucella abortus* to establish chronic infection in a mouse model [11]. The *bab_RS29915* gene encodes a putative lytic transglycosylase and its mutant showed reduced survival within RAW264.7 cells and was attenuated in a mouse model [12]. Among 38 novel virulence-related genes, the *bab_RS27735* gene was identified to be associated with *Brucella* virulence. The *bab_RS27735* gene is homologous to *aapJ*, but far away from the *aap* operon (*aapJQMP*) region in *B. abortus*, designated as *aapJ2* gene (Figure 1). The *aapJ2* gene encodes a putative amino acid ABC transporter substrate-binding protein AapJ2, which takes part in formation of an integral ABC transporter with other related proteins AapQ, AapM and AapP. The formed integral ABC transporter plays an important role in transportation and efflux of amino acids [13].

In this study, we investigated the role of the *aapJ2* gene in *B. abortus* virulence and found that the *aapJ2* is associated with *Brucella* intracellular survival and plays an important role in *Brucella* early infection in a mouse model. These data indicate that the amino acid ABC transporter plays an important role in the pathogenesis of *B. abortus* infections.

## Materials and methods

### Bacterial strains and growth conditions

*Brucella abortus* wild-type (WT) strain 2308 was obtained from the Chinese Veterinary Culture Collection Center (CVCC, Beijing, China) and routinely grown on tryptic soy broth (TSB, Difco™, BD BioSciences, Franklin Lakes, NJ, USA) or tryptic soy agar (TSA) at 37 °C under an atmosphere of 5% CO_2_. Manipulation of all live *B. abortus* strains were performed in a biosafety level 3 laboratory facility at the Chinese Academy of Agricultural Sciences. *Escherichia coli* strain DH5α (TIANGEN Biotech Co., Ltd., Beijing, China) was grown in Luria–Bertani medium. When appropriate, 100 μg/mL of ampicillin (Sigma-Aldrich Corporation, St. Louis, MO, USA) were added. All strains and plasmids used in this study are listed in Table 1.

**Table 1:** **Bacterial strains and plasmids used in this study**

Strains or plasmids	Descriptions	Sources or references
Bacterial strains		
*B. abortus* S2308	Wild-type strain; Smooth phenotype	CVCC
∆27735	*bab_RS27735* gene deletion mutant strain; Smooth phenotype	This study
*E. coli* DH5α	F^−^ φ80*lac*Z∆M15∆(*lac*ZYA-*arg*F)U169 *rec*A1 *end*A1 *hsd*R17(r_k_^−^, m_k_^+^) *pho*A *sup*E44 *thi*-1 *gyr*A96 *rel*A1 λ^−^	Invitrogen
Plasmids		
pSC	Amp^R^; pUC19 plasmid containing *SacB* gene	[10]

### Construction of the deletion mutant

Suicide plasmids were constructed using an overlap polymerase chain reaction (PCR) method, as we previously reported [11]. A 1030-bp upstream fragment and a 1026-bp downstream fragment of *bab_RS27735* were amplified by PCR using two primer pairs, 27735-UF/UR and 27735-DF/DR, respectively. Then, the two fragments were overlapped by PCR using the primers 27735-UF and 27735-DR. The overlap PCR product was cloned into the pSC plasmid. The recombinant suicide plasmid pSC-Δ27735 was extracted to construct the mutant. The primers used in this study are listed in Table 2.

**Table 2:** **Primers used in this study**

Primers	Sequences
27735-UF	GCTCTAGAGCTATCATGGCACCACGCAGGAAC (Xba I underlined)
27735-UR	GGCGGGGCGTACATCAGCCCCAGCAACGCACCCCAAACTG
27735-DF	CAGTTTGGGGTGCGTTGCTGGGGCTGATGTACGCCCCGCC
27735-DR	GCTCTAGAGCCTTTTCAATGAATGCACCGGC (Xba I underlined)
P1	CTGGGAGGAGGAACAATGAA
P2	CAGCCTGCTCAAGATCAACC
P3	GCGAAGGCGGAGCAATCT
RT-18500-F	CTGGTTGCTGGACTTCGTGT
RT-18500-R	GCTTGCCACGTCTTTCGATG
RT-20725-F	AGCAATCTCAAGGCAACGGA
RT-20725-R	GCATGCGAGATGGACGAAAC
RT-30275-F	AGTCGACATGTCTTCGCTGG
RT-30275-R	GATCAGGTCATGTCCCACGG
RT-30280-F	AGGCGTAACGGATGTCTTGG
RT-30280-R	CTGCGCGCTGAAAGAGATTG
RT-26600-F	ACCATGCTTGATTCGCTTGC
RT-26600-R	GTCAACTGATTGCGATGGGC
RT-26930-F	AAGCCTGCAAGACCTCAACC
RT-26930-R	CTGAAGCTTTGGGTCTGCCT
RT-31530-F	GCCGCTCAACGAAATCATCC
RT-31530-R	GTCACGATGCGCTGAAGAAC
RT-27100-F	CTCGGCATGCAGAACAATCC
RT-27100-R	TCGCGGTCCGTCTTGTTATC
RT-31650-F	GATTCCACACGGCCAACAAC
RT-31650-R	GTGGCGTTGAACATGAGCTG
RT-29200-F	GACGGGGCGGAGATTGTTTA
RT-29200-R	CGCAGTTCGGTTTGTTCCAG
RT-28255-F	ATGACCTATGCGGTTCAGGC
RT-28255-R	CTCGATCTTTGGGCGGAACA
RT-18900-F	CGGCAACACCTTTCAACTCC
RT-18900-R	CCTTTCGTCCACGCAGATCA
RT-30140-F	CCAGCTTCATGTCGCTTCCT
RT-30140-R	AGAATAAGCTGGTCGCGGAA
RT-18875-F	CTGGAGTACGGCCAGTGAAG
RT-18875-R	ATCAGGTTGACGAGCCTGTG
RT-27050-F	ACAGCAACATCATGCGGGTA
RT-27050-R	ATATGCTGGTCGTTCAGGGC
RT-23095-F	ACCAGATCAATCTCGTGGGC
RT-23095-R	GCATGAACGCGAAAAACCCT
RT-31525-F	AACAGGTCGAATCACGGCTC
RT-31525-R	ATCCGATGTTTTGCGGCTGA
RT-22085-F	AAAATTGCCGAACAGGCCAC
RT-22085-R	GGCCTTGTTGCCGTATTCAC
RT-27055-F	ACGGTTATGGCTTCCGGTTC
RT-27055-R	ATCTTGACGGTTGCTCCCAG
RT-GAPDH-F	GACATTCAGGTCGTCGCCATCA
RT-GAPDH-R	TCTTCCTTCCACGGCAGTTCGG

The Δ27735 mutant was constructed by allelic replacement using a two-step strategy, as we previously described [11]. Briefly, the bacterial cells were prepared through two washes with ice-cold sterile water, and the suicide plasmid pSC-Δ27735 (0.5–1.0 μg) was transformed into the pretreated bacterial cells by electroporation. The single exchanged recombinants were selected by plating on TSA containing ampicillin, and then colonies were inoculated into TSB without antibiotics. The second exchanged recombinants were selected by plating on TSA containing 5% sucrose. All colonies were selected and verified by PCR amplification.

### Determination of bacterial growth curve

Bacterial growth was measured at optical density 600 nm (OD_600_). The WT strain and the Δ27735 mutant were cultured in TSB to generate growth curves, as described elsewhere [12]. Freshly cultured bacteria were diluted and the value of OD_600_ was adjusted to 1.0. Then, 1 mL of the bacterial suspension was inoculated into 100 mL of TSB and cultured at 37 °C at 200 rpm. The OD_600_ absorbance of aliquots was measured every 4 h.

### Stress resistance assay

H_2_O_2_ was used to determine sensitivity of the Δ27735 mutant to oxidative stress and polymyxin B was used to test its sensitivity to cationic bactericidal peptides. The WT strain and the mutant were cultured to mid-logarithmic phase (the value of OD_600_ ≈ 1.0) in TSB medium, and then the bacterial suspension was diluted with PBS and adjusted to a concentration to 4 × 10^5^ colony-forming units (CFU)/mL. Afterward, 50 μL of bacterial suspension was mixed with 50 μL of the appropriate reagent. H_2_O_2_ was used to determine sensitivity to oxidative stress and added at final concentrations of 0.5, 1 or 2 mM. Polymyxin B at concentrations of 25, 50 or 100 μg/mL was used to test sensitivity to cationic bactericidal peptides. In all tested groups, a negative-control group was introduced by adding 50 μL of PBS to the same bacterial suspension. The bacterial survival percentages were calculated as: (CFU obtained from bacteria treated with different factors/CFU obtained from bacteria in PBS) × 100%. The results are expressed as the mean percentage of triplicate samples ± standard deviation from one independent experiment.

Acid peptone water was used to assess the acid tolerance of the mutant [14]. A bacterial suspension of the WT and the mutant strain was diluted to 2 × 10^7^ CFU/mL in peptone water with pH of 7.3, 5.5 or 4.5. After 1 h of incubation at 37 °C, cells were serially diluted and plated on TSA to determine the number of CFU. The percentage of surviving bacteria pH at 5.5 and 4.5 was calculated with respect to CFU obtained from bacteria incubated in peptone water at pH 7.3.

### LPS extraction and silver staining

The WT strain and the Δ27735 mutant were cultured to the exponential phase in TSB. The bacterial cells were collected by centrifugation, and LPS was extracted using an LPS Extraction Kit (iNtRON, Seoul, Korea). Samples were loaded on 12.5% polyacrylamide gels for SDS-PAGE and a silver staining assay was performed as previously described [12].

### Cell infection assay

RAW 264.7 macrophages were used to assess the ability of the Δ27735 mutant to survive intracellularly. The experiment was performed as previously reported [14, 15]. Briefly, cells were seeded in 24-well plates and grown in Dulbecco’s Modified Eagle Medium (DMEM) (Hyclone™; GE Healthcare, Chalfont St. Giles, UK) supplemented with 10% fetal bovine serum (FBS) (Gibco^®^; Invitrogen Corporation, Carlsbad, CA, USA) at 37 °C under an atmosphere of 5% CO_2_ for 24 h. The cell monolayer was washed twice with DMEM and infected with the WT strain or the Δ27735 mutant at a multiplicity of infection (MOI) of 200. Bacteria were centrifuged onto the cells at 400 × *g* for 5 min and the cells were then incubated at 37 °C for 1 h. Non-adherent bacteria were removed by rinsing the wells twice with DMEM. To kill extra-cellular bacteria, the cells were incubated with DMEM containing gentamicin (100 μg/mL) for an additional 1 h and washed twice with DMEM. Afterward, the medium was replaced with DMEM containing 2% FBS and 20 μg/mL of gentamicin. At 2, 8, 24 and 48 h post-infection (pi), the macrophages were lysed with 0.2% Triton X-100 in sterile water and the live bacteria were enumerated on TSA plates. All assays were performed in triplicate and repeated at least three times. The results are presented as the averages of triplicate infection samples ± standard deviation at one independent experiment.

### Immunofluorescence assay

RAW 264.7 cells were cultured on 15-mm glass coverslips (Thermo Scientific, Waltham, MA, USA) in 24-well plates and infected with the WT strain or the Δ27735 mutant at an MOI of 200, as described above. At 4 and 24 h pi, the cells were washed twice with PBS and fixed overnight in 4% (w/v) paraformaldehyde at 4 °C. Fluorescence staining and a *Brucella* co-localization assay with lysosomes were performed as described in our previous report [11]. Rabbit anti-*Brucella* polyclonal antibody (1:500 dilution) was used to track intracellular bacteria. Rat LAMP-1 (lysosome associated membrane protein 1) monoclonal antibody (1:1000 dilution; Abcam, Cambridge, UK) was used to track the lysosomes. Goat anti-rabbit Alexa Fluor 488 and goat anti-rat Alexa Fluor 555 (Thermo Fisher Scientific, Waltham, MA, USA) were used as secondary antibodies at dilutions of 1:1000. The cells were observed under laser scanning confocal microscope (Nikon D-Eclipse C1, Tokyo, Japan) with 100× oil immersion objective. Images were saved in TIFF format and imported to Adobe Photoshop CS4 (Adobe Systems Incorporated, San Jose, CA, USA), where they were merged using RGB format. To determine the percentage of bacteria positive for the lysosome marker LAMP-1, 100 intracellular bacteria were counted randomly. Assays were performed in triplicate.

### Mouse infection assay

To investigate bacterial virulence, three groups of the WT strain, the Δ27735 mutant and the blank control were designed. Each group included thirty 6-week-old female BALB/c mice, which were tested at five timepoints. The WT strain and the mutant were intraperitoneally inoculated into mice at 1 × 10^5^ CFUs. The mice in the blank group were intraperitoneally inoculated with PBS. At 2, 4, 6, 9 or 12 weeks pi, six mice in each group were euthanized. From 5 of these mice the spleens were collected, weighed, and homogenized in 5 mL of 0.2% (v/v) Triton X-100 PBS solution. Then, 100-μL aliquots were used for tenfold serial dilutions plated on TSA to determine the number of bacterial CFUs. From one mouse per group both the spleen and liver were collected and fixed in 4% (v/v) formaldehyde for histopathological examination. Besides, peripheral blood samples of the infected mice were collected to determine the levels of TNF-α and IL-12p40 using ELISA kits (Yaoyun, Shanghai, China). The peripheral blood samples from PBS inoculated mice were used as the blank control.

### RNA extraction, RNA-seq analysis and quantitative real-time PCR (qPCR)

Total RNA was extracted from the WT strain and the Δ27735 mutant using the RiboPure™ Bacteria kit (Ambion, Carlsbad, CA, USA). RNA-seq analysis was performed by the Beijing Genomics Institute (BGI, Wuhan, China). For qPCR, RNA was reverse transcribed into cDNA using the PrimeScript RT reagent kit (Takara Bio, Inc., Shiga, Japan) at 37 °C for 20 min, then at 85 °C for 10 s for the cDNA templates. qPCR was performed using 2× GoTaq qPCR master mix (Promega Corporation, Madison, WI, USA). Reactions were carried out on a Mastercycler ep Realplex system (Eppendorf AG, Hamburg, Germany) at 95 °C for 2 min, followed by 40 cycles at 95 °C for 15 s and 60 °C for 1 min. For each gene, PCR was performed in triplicate and relative transcription levels were determined by the 2^−ΔΔCt^ method using glyceraldehyde phosphate dehydrogenase (*gapdh*) as an internal control for data normalization. All primers used for qPCR are listed in Table 2.

### Statistical analysis

Data were imported into GraphPad Prism 6.0 software (GraphPad Software, Inc., La Jolla, CA, USA) for analysis. Statistical significance was determined using the unpaired or two-tailed Student’s *t* test. For group analysis, two-way ANOVA followed by Holm-Sidak’s multiple test was used. A probability (*p*) value of < 0.05 was considered statistically significant.

## Results

### The Δ27735 mutant was constructed successfully without phenotype changes

**Figure 2:**
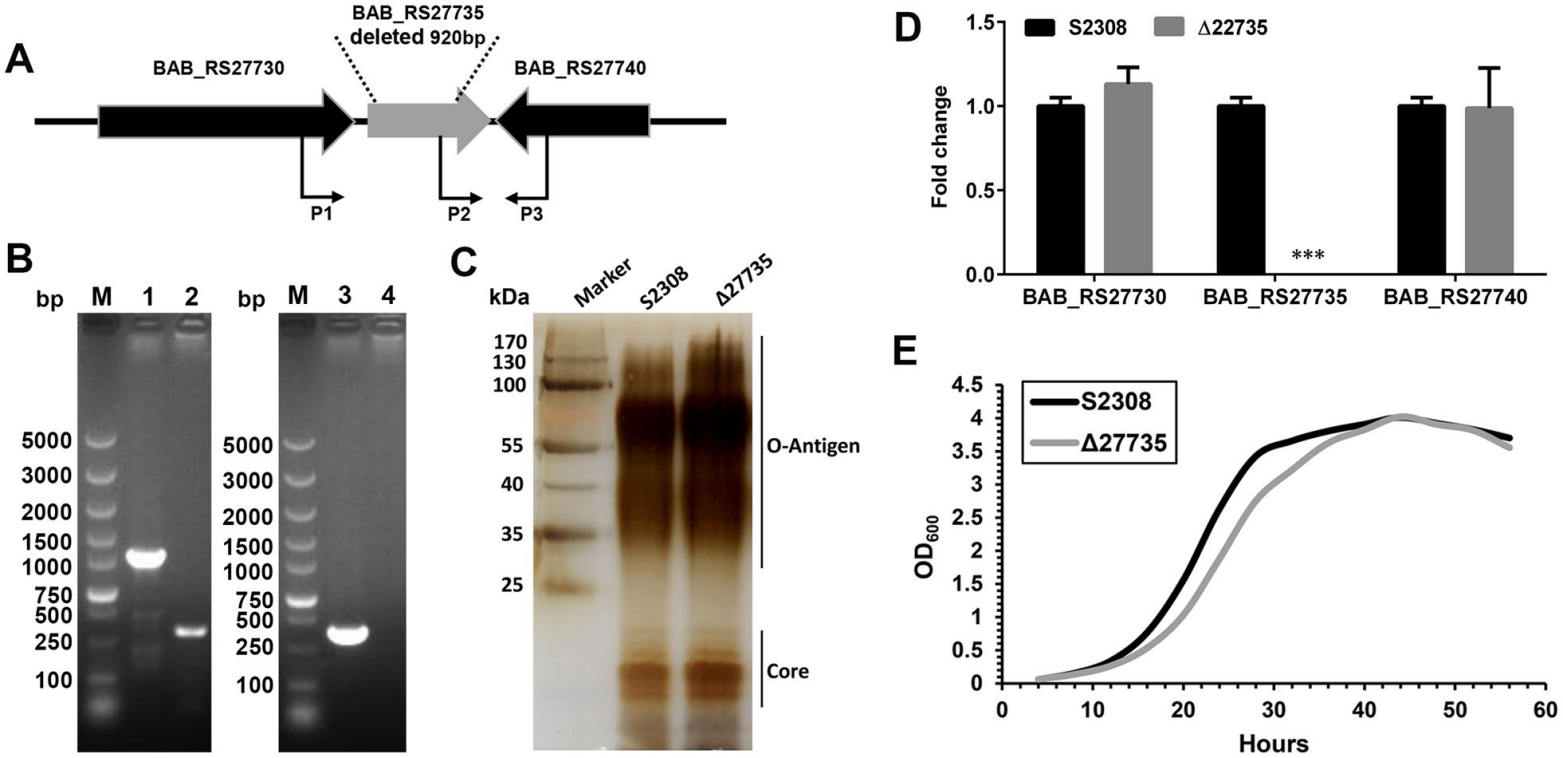
**A** ***bab_RS27735*** **deletion mutant was successfully constructed without phenotype changes.** Genetic organization of the *bab_RS27735* locus and position of the primers designed to identify the Δ27735 mutant (**A**). Identification of the Δ27735 mutant by polymerase chain reaction (PCR) using the outer primers P1/P3 (left panel) and the crossover primers P2/P3 (right panel). Lane 1 and 3 refer to the wild-type (WT) strain; lane 2 and 4 refer to the Δ27735 mutant (**B**). Extraction and silver staining of lipopolysaccharide (**C**). Determination of the transcriptional levels of the flanking genes by quantitative real-time PCR (**D**). Determination of growth curve in tryptic soy broth (**E**). Statistical significance was determined using the unpaired Student’s *t* test. ***p < 0.001.

The *bab_RS27735* gene encodes a putative amino acid ABC transporter substrate-binding protein in *B. abortus*, and its flanking genes *bab_RS27730* and *bab_RS27740* encode a hypothetical protein and D-aminopeptidase, respectively (Figure 2A). To investigate the role of *bab_RS27735* in *Brucella* virulence, a *bab_RS27735* deletion strain was constructed with the deletion of a 960-bp fragment, which resulted in the loss of 94% of the open reading frames. The mutant was confirmed by PCR (Figure 2B). The P1 and P3 primers were designed to amplify a 1295-bp fragment containing the entire *bab_RS27735* gene of the WT strain, but a truncated 341-bp fragment was amplified from the mutant due to the loss of a 960-bp fragment of the *bab_RS27735* gene (Figure 2B). The P2 and P3 primers were designed by crossing the *bab_RS27735 and bab_RS27740* genes of the WT strain. A 379-bp fragment was amplified, but in the mutant, no band was shown (Figure 2B). Together, these results confirmed successful construction of the Δ27735 mutant.

*Brucella* is reported to tend to randomly lose the O-antigen of LPS during mutant construction, which is an important interference factor for assessing *Brucella* virulence [16, 17]. To avoid this situation, the LPS of the Δ27735 mutant was extracted and analyzed with silver staining. The results showed no changes in the patterns of LPS between the Δ27735 mutant and its WT strain (Figure 2C). To further confirm whether the *bab_RS27735* deletion had a polar effect on the transcription of the flanking genes *bab_RS27730* and *bab_RS27740*, qPCR was performed, which revealed no effect on gene expression (Figure 2D). Besides, a growth curve was constructed to assess the impact of the *bab_RS27735* deletion on the growth of *B. abortus*. As shown in Figure 2E, as compared to the WT strain cultured in TSB, growth of the Δ27735 mutant was slightly defective in the exponential phase, but the cells reached a similar stationary phase after 40 h of incubation.

### The *bab_RS27735* gene is not associated with *Brucella* resistance to oxidative stress, bactericidal peptides, or low pH

**Figure 3:**
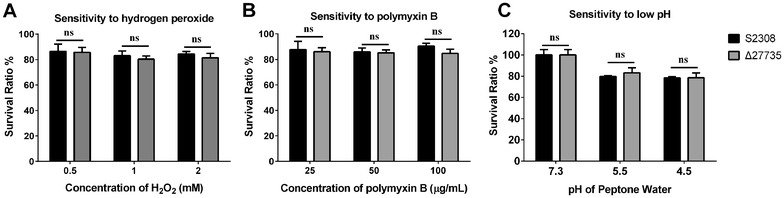
**Determination of bacterial sensitivity to hydrogen peroxide, polymyxin B, and low pH.** There were no differences in sensitivities to hydrogen peroxide (**A**), polymyxin B (**B**), or low pH (**C**) between the WT strain and the Δ27735 mutant. Statistical significance was determined using two-tailed Student’s *t* test. ns: not significant (*p* > 0.05).

To further confirm the sensitivity of the Δ27735 mutant to oxidative stress, bactericidal peptide, and low pH, the ability of the mutant to resist hydrogen peroxide, polymyxin B, and low pH was assessed. Both the mutant and the WT strain showed a similar survival rate of 80–85% under different concentrations of hydrogen peroxide, suggesting that the *bab_RS27735* gene was not associated with *Brucella* resistance to oxidative stress (Figure 3A). The sensitivity of the mutant to polymyxin B was not obviously changed in comparison with the WT strain, revealing that *bab_RS27735* gene was not involved in *Brucella* resistance to bactericidal peptides (Figure 3B). The results of the acid tolerance test showed that the survival rates of the mutant and the WT strain were both ~80%, with no significant difference. These results confirmed that the *bab_RS27735* gene is not associated with the ability of *Brucella* resistance to oxidative stress, bactericidal peptides, or low pH.

### The *bab_RS27735* gene plays a role in *Brucella* intracellular survival at an early stage, but is not involved in preventing lysosome fusion

**Figure 4:**
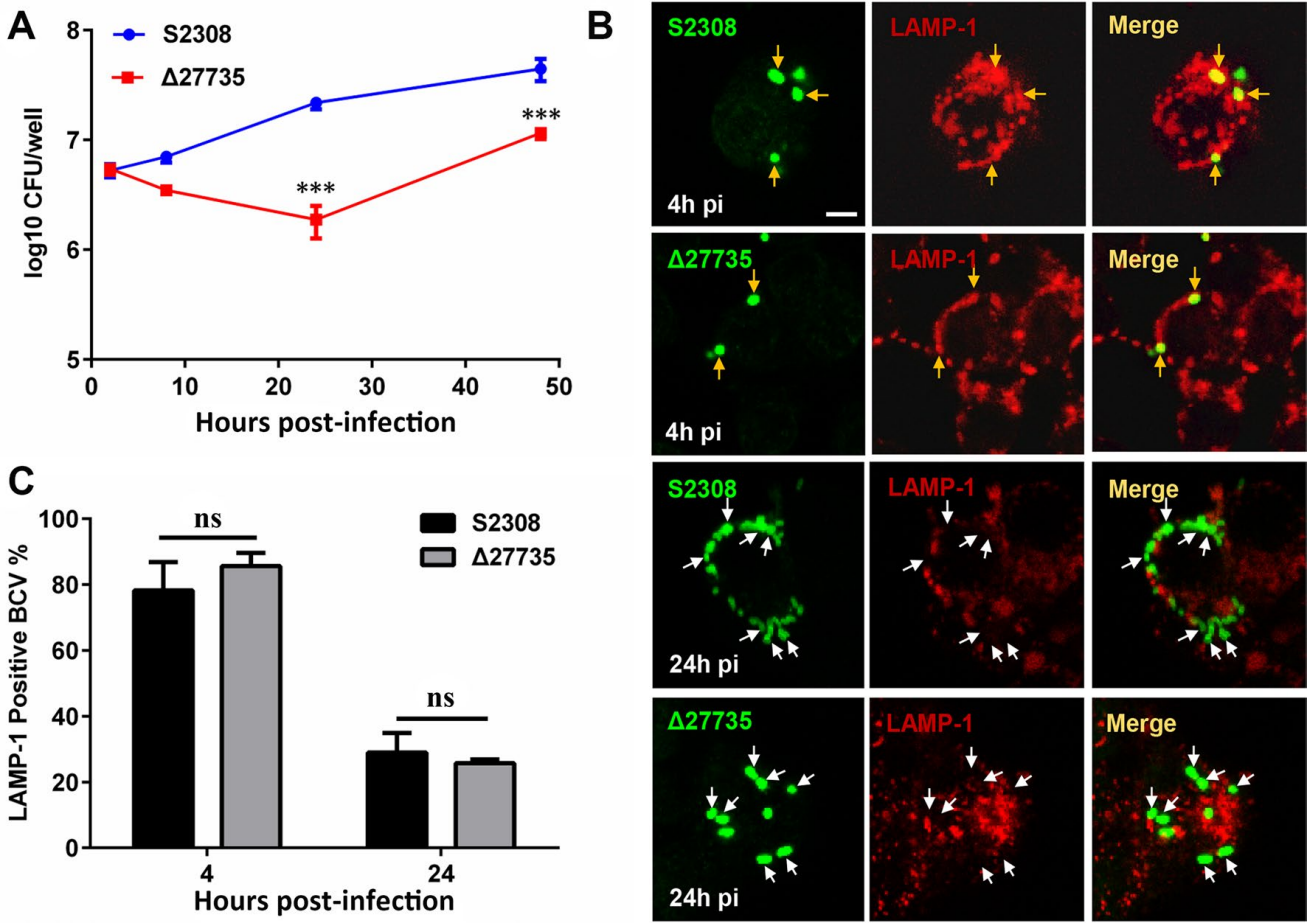
**The Δ27735 mutant reduced intracellular survival, but intracellular trafficking was unchanged.** Intracellular survival of the WT strain and the Δ27735 mutant within RAW264.7 cells was determined at 2, 8, 24 and 48 h post-infection (pi) (**A**). Representative images of lysosome associated membrane protein-1 (LAMP-1) -positive (yellow arrows) and -negative (white arrows) *Brucella* -containing vacuoles (BCVs) at 4 and 24 h pi in the WT strain and the Δ27735 mutant in RAW264.7 cells (**B**). Determination of LAMP-1 -positive BCVs at 4 and 24 h pi in the WT strain and the Δ27735 mutant (**C**). Statistical significance was determined using two-way ANOVA followed by Holm-Sidak’s multiple test. ***p < 0.001; ns: not significant (*p* > 0.05).

Intracellular survival is an important manifestation of *Brucella* virulence. Therefore, to assess the role of *bab_RS27735* in bacterial virulence, the intracellular survival of the Δ27735 mutant and the WT strain within RAW264.7 cells was determined. The results showed that intracellular survival of the mutant was significantly reduced at 24 and 48 h pi, as compared to the WT strain. This difference was not due to the ability to invade the host cells, because at 2 h pi, similar CFUs were recovered from the mutant- and WT strain-infected cells. Interestingly, the mutant obviously increased survival inside macrophages at 48 h pi, as compared to that at 24 h pi. These data indicated that the mutant can survive within cells, but the time needed for replication within macrophages was prolonged (Figure 4A). To this end, we evaluated the ability of the mutant to evade fused lysosomes by assessing the co-localization of *Brucella* and LAMP-1, a representative marker of late endosome/lysosome fusion. Both the mutant and WT strains were co-localized with LAMP-1 of about 80% at 4 h pi, and about 20–25% at 24 h pi (Figure 4B). In other words, about 70–80% of both strains successfully excluded LAMP-1 at 24 h pi (Figure 4C). These results suggest that survival of the Δ27735 mutant within macrophages was reduced, but not because of the defective intracellular trafficking of *Brucella*.

### The Δ27735 mutant is attenuated at early stage of infection in mouse model

**Figure 5:**
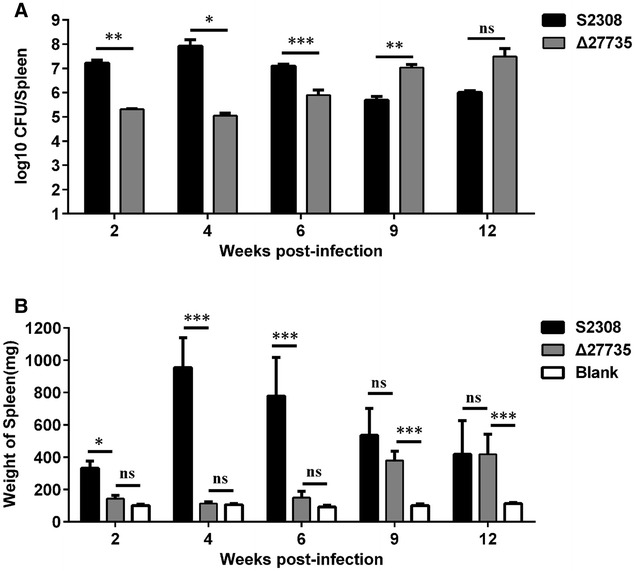
**The Δ27735 mutant was attenuated at the early stage of infection in a mouse model.** The bacteria were recovered from spleens infected by the WT strain and the Δ27735 mutant (**A**). Splenomegaly was assessed by weighing the infected spleen (**B**). Statistical significance was determined using two-way ANOVA followed by Holm-Sidak’s multiple test. *p < 0.05; **p < 0.01; ***p < 0.001; ns: not significant.

In further study, we evaluated the function of the *bab_RS27735* gene in *Brucella* virulence using the directional deletion mutant strain Δ27735 in a mouse model over a longer period than the time (5 weeks pi) we used in the STM screening. At the early stage of infection (2 weeks pi), the Δ27735 mutant exhibited significantly reduced virulence, as compared to the WT strain, suggesting that the *bab_RS27735* gene is necessary for early infection by *Brucella* (Figure 5A). At the peak time of *Brucella* infection (4 weeks pi), the WT strain reached a high bacterial load in the spleen of infected mice of about 10^8^ CFU, whereas the bacterial load of the mutant-infected spleen was only about 10^5^ CFU. At 6 weeks pi, although the bacterial load of the WT-infected spleen had decreased, the load in the mutant-infected spleen had increased, as compared to measurements at 2 and 4 weeks pi. However, the load of the mutant strain was significantly lower compared to that of the WT strain at the same stage. It is worth noting that the bacterial load of the mutant continued to increase at 8 and 12 weeks pi, and obviously exceeded that of the WT strain. Splenomegaly was also assessed at different time points pi at the early (2 weeks pi) and the peak infection (4–6 weeks pi) stages, there was no obvious enlargement of the spleen infected by the mutant, as compared to the blank control group, but the spleens infected by the WT strain had swollen significantly at both stages of infection (Figure 5B). At 8 and 12 weeks pi, there was no difference in the extent of splenomegaly between the spleens infected with the mutant and WT strains, as both strains induced significant swelling of the spleen, as compared to the blank group (Figure 5B). These data revealed that *bab_RS27735* plays a significant role in *Brucella* virulence at the early stage, but not the late stage of infection in a mouse model.

### The Δ27735 mutant induced less cytokine release in the infected mice, but promoted similar development of pathological lesions, as the WT strain

**Figure 6:**
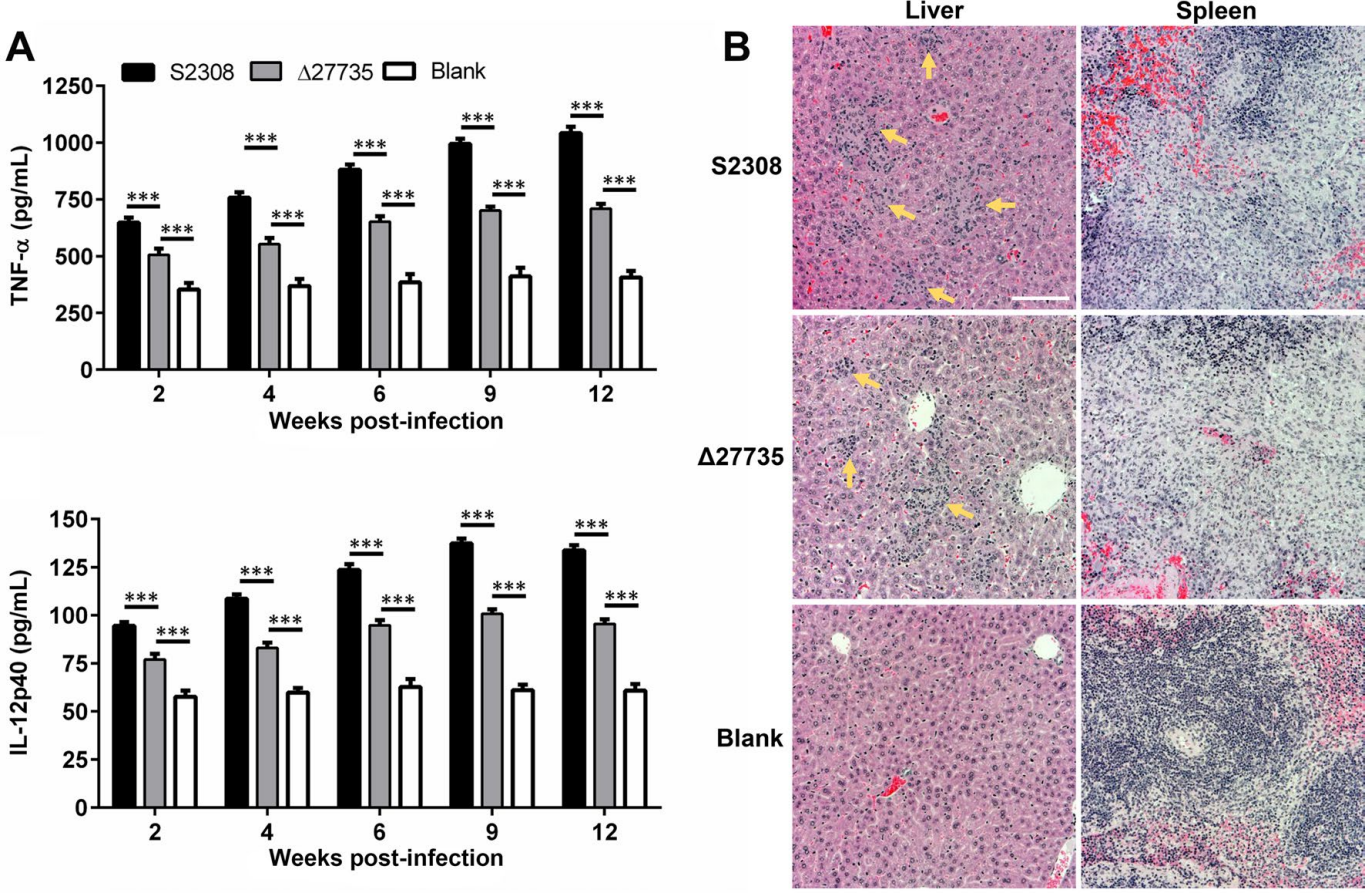
**A mouse infected with the Δ27735 mutant released significantly higher levels of cytokines into the peripheral blood and induced the development of pathological lesions in the spleen and liver.** The concentrations of TNF-α and IL-12p40 in the peripheral blood of mice infected with the WT strain and the Δ27735 mutant were determined at 2, 4, 6, 9, and 12 weeks pi (**A**). Histopathological analysis of the liver and spleen infected with the WT strain or the Δ27735 mutant at 12 weeks pi by hematoxylin–eosin staining (200×, **B**). Statistical significance was determined using two-way ANOVA followed by Holm-Sidak’s multiple test. ***p < 0.001.

As described earlier, the Δ27735 mutant cannot effectively survive within macrophages and had reduced virulence at the early stage, but not the late stage, of infection. Besides, splenomegaly was similar at the late stage between the mutant and the WT strain, so we hypothesized that there were differences in the inflammatory responses associated with splenomegaly in mice infected with the mutant vs. the WT strain. To confirm this hypothesis, we evaluated the inflammatory responses in mice infected with the mutant vs. WT strain. The results showed increased TNF-α and IL-12p40 release in the peripheral blood during the entire infection process in mice infected with either the Δ27735 mutant of the WT strain, as compared to the blank group (Figure 6A). However, at the early and peak infection stage (2–6 weeks pi), the Δ27735 mutant hardly induced splenomegaly (Figure 5B). Besides, as compared to the WT strain, the mutant induced less TNF-α and IL-12p40 release in the peripheral blood of the infected mice throughout the entire infection process (Figure 6A). Histopathological examination showed that small granulomas were induced in the livers infected by the WT strain and the mutant by the aggregation of lymphocytes at 12 weeks pi (Figure 6B). In the infected spleens of the mutant and WT infected groups, the boundaries of white and red pulp were ambiguous, accompanied by an abundance of infiltrating lymphocytes and lymphohistiocytosis. These data suggested that the Δ27735 mutant has strong residual virulence, although the bacterial load of the mutant-infected spleen was reduced at the early stage of infection. Nonetheless, the outcome of disease caused by the mutant at the late infection stage was similar to that of the WT strain.

### The *bab_RS27735* gene deletion changed *Brucella* diverse genes expression

To further investigate the role of *bab_RS27735* in *Brucella* virulence, RNA-seq was performed to analyse the differential expression of genes in the *bab_RS27735* mutant compared to the WT strain. In total, 3087 transcripts were detected in the *Brucella* strains. Of these, 19 genes were selected for further investigation as main differential expression genes, because the transcriptional level of these genes in the mutant was increased or decreased more than twofold changes in comparison with the WT strain. In a further study, qPCR was carried out to verify the 19 genes expression in the mutant. The results showed that the expression profiles of 19 genes had changed (Table 3). Among these, expression of seven genes in the mutant was up-regulated by more than 1.5-fold, while expression of none of the genes was down-regulated by more than 1.5-fold, as compared to the WT strain. Of the seven up-regulated genes, *bab_RS30275* (1.66 ± 0.44-fold) is an extracellular ligand-binding receptor involved in amino acid transport and *bab_RS30280* (1.77 ± 0.23-fold) encodes NADPH quinone oxidoreductase, which is associated with bacterial energy production and conversion. The *bab_RS26930* (1.69 ± 0.17-fold) and *bab_RS31530* (1.53 ± 0.25-fold) genes encode flagellar related proteins. However, the function of the *bab_RS18900* (1.84 ± 0.56-fold)*, bab_RS30140* (1.71 ± 0.38-fold), and *bab_RS18875* (1.70 ± 0.36-fold) genes remain unclear. These data suggest that the *bab_RS27735* gene deletion changed the expression of various *Brucella* genes.

**Table 3:** **The differential expression genes in the Δ27735 mutant compared to the WT strain**

Gene locus	Gene products	Regulation	2^−∆∆Ct^ ± SD
Replication, recombination and repair			
*bab_RS18500*	Transposase	Up	1.13 ± 0.11
*bab_RS20725*	Transposase, is4 family	Down	0.81 ± 0.05
Amino acid transport and metabolism			
*bab_RS30275*	Extracellular ligand-binding receptor	Up	1.66 ± 0.44*
Energy production and conversion			
*bab_RS30280*	NADPH quinone oxidoreductase	Up	1.77 ± 0.23*
Post-translational modification, protein turnover, and chaperones			
*bab_RS26600*	Heat shock protein	Up	1.22 ± 0.13
Cell motility			
*bab_RS26930*	Flagellar motor switch protein	Up	1.69 ± 0.17*
*bab_RS31530*	Flagellar biosynthesis protein	Up	1.53 ± 0.25*
*bab_RS27100*	Flagellar L-ring protein	Down	0.82 ± 0.16
Transcription			
*bab_RS31650*	AraC family transcriptional regulator	Down	0.73 ± 0.12
*bab_RS29200*	IclR family transcriptional regulator	Down	0.69 ± 0.07
Defense mechanisms			
*bab_RS28255*	Type I restriction DNA specific modify protein	Down	0.73 ± 0.07
Function unknown			
*bab_RS18900*	Hypothetical protein	Up	1.84 ± 0.56*
*bab_RS30140*	LmbE family	Up	1.71 ± 0.38*
*bab_RS18875*	Putative Gene transfer agent	Up	1.70 ± 0.36*
*bab_RS27050*	Hrp-dependent Type III effector	Up	1.26 ± 0.13
*bab_RS23095*	Predicted membrane protein (DUF2306)	Up	1.23 ± 0.20
*bab_RS31525*	Hypothetical protein	Up	1.20 ± 0.35
*bab_RS22085*	Hypothetical protein	Down	0.76 ± 0.23
*bab_RS27055*	Hypothetical protein	Down	0.76 ± 0.12

Statistical significance was determined using two-tailed Student’s *t* test.

*p < 0.05.

## Discussion

*Brucella* is a facultative intracellular bacterium that invades and then replicates in the host cell, which is crucial for its virulence [18]. Amino acid utilization by intracellular *Brucella* is thought to be associated with its virulence, because the expression of *Brucella* genes involved in amino acid metabolism is induced intracellularly [19]. In our previous study, we identified eight *Brucella* virulence-related genes associated with amino acid transport and metabolism by STM screening (data unpublished), such as glycine dehydrogenase, histidinol-phosphate amino transferase, and histidinol dehydrogenase. Of these genes, *bab_RS27735*, which encodes a putative amino acid ABC transporter substrate-binding protein, was identified as a *Brucella* virulence-related gene. In this study, we further investigated the role of *bab_RS27735* in *Brucella* virulence.

The BAB_RS27735 protein is homologous with the AapJ protein, showing 56% identity with *Sinorhizobium fredii*. This protein is widely distributed in prokaryotic organisms and is reportedly associated with l-amino acid uptake and the efflux of solutes in *Rhizobium leguminosarum* [20]. In this study, the *BAB_RS27735* deletion mutant showed reduced intracellular survival within RAW264.7 cells. Interestingly, at 48 h pi, intracellular survival was partially restored in the mutant. This result is in line with the co-localization of the mutant and lysosome marker LAMP-1, revealing that the *bab_RS27735* mutant can successfully inhibit lysosome fusion within host cells, which is a crucial step in *Brucella* intracellular trafficking [21]. This indicates that the reduced survival of the mutant is not due to the defect of intracellular trafficking, but more likely due to its inability to transport and utilize amino acids, because under in vitro cultural conditions, growth of the mutant was decreased. In a mouse model, the mutant was attenuated at the early stage of infection (2–6 weeks pi), but virulence was restored at the late stage of infection (9–12 weeks pi), suggesting that virulence was reduced in the mutant at the early stage of infection due to killing by the host innate immunity. However, the in vitro data did not reveal an increased sensitivity of the mutant strain to oxidative stress, bactericidal peptides, or low pH. In addition, the mutant induced the development of severe splenomegaly and histopathological lesions at the late stage of infection, as compared to the WT strain, indicating that the virulence of the mutant was delayed, rather than attenuated, which might have been caused by defective amino acids transport and utilization. However, in this study, the ability of the *bab_RS27735* mutant to uptake amino acids was not assessed. Besides, *B. abortus* has two copies of the AapJ protein, AapJ1 and AapJ2. Deletion of AapJ2, which is encoded by *bab_RS27735* in *B. abortus*, might be compensated by the truncated AapJ1 protein. This compensatory effect may delay the ability of the *bab_RS27735* mutant to cause disease. On the other hand, in *R. leguminosarum*, Aap is an active uptake system that also affects the efflux of a broad range of amino acids. An Aap mutant prevented the efflux of intracellular amino acids, while overexpression increased the efflux rates [20]. However, it remains unclear whether efflux of intracellular amino acids is important for *Brucella* replication within the host cell, thus further experiments are warranted. Moreover, deletion of the *bab_RS27735* gene in *B. abortus* up-regulated the expression of several genes that may indirectly influence the ability of the host immune system to recognize the mutant by up-regulation of the flagellar synthesis-related genes *bab_RS26930* and *bab_RS31530*. The flagellum is an important bacterial pathogen-associated molecular pattern that is well recognized by the host innate immune system [22]. However, up-regulation may enhance exposure to the host immune system, resulting in the elimination of the *bab_RS27735* mutant. Several factors may thus contribute to the reduced virulence of the *bab_RS27735* mutant, including its reduced survival within macrophages, its defected amino acid transportation or efflux, and its delayed activation of the host inflammation response. The synergy of all factors may determine the infection state of the *bab_RS27735* mutant at the early stage in a mice model.

Overall, in this study, we identified a putative amino acid ABC transporter substrate-binding protein, AapJ2, which is encoded by the *bab_RS27735* gene. Although AapJ2 is far away from the *aap* operon region, this gene is necessary for *Brucella* survival within macrophages, as its mutant had reduced virulence at the early stage of infection in a mouse model. The role of *bab_RS27735* in virulence in the natural host requires further studies.

## Abbreviations

WT: wild-type
PCR: polymerase chain reaction
TSB: tryptic soy broth
TSA: tryptic soy agar
DMEM: Dulbecco’s Modified Eagle Medium
FBS: fetal bovine serum
pi: post-infection
STM: signature-tagged mutagenesis
CFU: colony-forming unit
qPCR: quantitative real-time PCR
